# Volumetric analysis of the piriform cortex in temporal lobe epilepsy

**DOI:** 10.1016/j.eplepsyres.2022.106971

**Published:** 2022-09

**Authors:** Sabahat Iqbal, Jose E. Leon-Rojas, Marian Galovic, Sjoerd B. Vos, Alexander Hammers, Jane de Tisi, Matthias J. Koepp, John S. Duncan

**Affiliations:** a UK National Institute for Health Research University College London Hospitals Biomedical Research Centre, and Department of Clinical and Experimental Epilepsy, UCL Queen Square Institute of Neurology, London, United Kingdom; b Epilepsy Society MRI Unit, Chalfont Centre for Epilepsy, Chalfont St Peter, Buckinghamshire, United Kingdom; c Facultad de Ciencias Médicas de la Salud y de la Vida, Escuela de Medicina, Universidad Internacional del Ecuador, Quito, Ecuador; d Department of Neurology, Zurich University Hospital, Zurich, Switzerland; e Centre for Medical Image Computing (CMIC), Department of Computer Science, University College London, United Kingdom; f Neuroradiological Academic Unit, UCL Queen Square Institute of Neurology, London, United Kingdom; g School of Biomedical Engineering and Imaging Sciences, Kings College, London, United Kingdom; h Kings College London & Guys and St Thomas’ PET Centre at St. Thomas’ Hospital, United Kingdom

**Keywords:** Piriform cortex, Hippocampus, Temporal lobe epilepsy

## Abstract

The piriform cortex, at the confluence of the temporal and frontal lobes, generates seizures in response to chemical convulsants and electrical stimulation. Resection of more than 50% of the piriform cortex in anterior temporal lobe resection for refractory temporal lobe epilepsy (TLE) was associated with a 16-fold higher chance of seizure freedom. The objectives of the current study were to implement a robust protocol to measure piriform cortex volumes and to quantify the correlation of these volumes with clinical characteristics of TLE. Sixty individuals with unilateral TLE (33 left) and 20 healthy controls had volumetric analysis of left and right piriform cortex and hippocampi. A protocol for segmenting and measuring the volumes of the piriform cortices was implemented, with good inter-rater and test-retest reliability. The right piriform cortex volume was consistently larger than the left piriform cortex in both healthy controls and patients with TLE. In controls, the mean volume of the right piriform cortex was 17.7% larger than the left, and the right piriform cortex extended a mean of 6 mm (Range: −4 to 12) more anteriorly than the left. This asymmetry was also seen in left and right TLE. In TLE patients overall, the piriform cortices were not significantly smaller than in controls. Hippocampal sclerosis was associated with decreased ipsilateral and contralateral piriform cortex volumes. The piriform cortex volumes, both ipsilateral and contralateral to the epileptic temporal lobe, were smaller with a longer duration of epilepsy. There was no significant association between piriform cortex volumes and the frequency of focal seizures with impaired awareness or the number of anti-seizure medications taken. Implementation of robust segmentation will enable consistent neurosurgical resection in anterior temporal lobe surgery for refractory TLE..

## Highlights

• Right piriform cortex is larger than left.
• Right piriform cortex extends more anteriorly into frontal lobe.
• Piriform cortex is smaller in those with hippocampal sclerosis.
• Piriform cortex volumes are smaller with a longer duration of epilepsy.

## 1 Introduction

In adults, temporal lobe epilepsy (TLE) is the most prevalent focal seizure disorder (Gonçalves et al., 2005). TLE with evidence of hippocampal sclerosis (HS) is highly amenable to epilepsy surgery (Vakharia et al., 2018). In a randomised control trial (Wiebe et al., 2001), the rate of seizure cessation was 64% for patients undergoing surgery for TLE. A more recent large cohort study established that the rate of seizure freedom at 5 years in patients who underwent anterior temporal resection was 55% (95% CI 51–60) (de Tisi et al., 2011). Thus, there is an urgent need to identify the factors which can be targeted to increase the present rate of post-surgical seizure freedom in these subsets of patients with TLE.

The current surgical practice for anterior temporal lobe resection involves the resection of the limbic regions of the medial temporal lobe i.e. amygdalohippocampal complex (Bertram, 2009). The incomplete removal of a critical area of epileptogenesis might be the underlying cause of ongoing seizures in patients with TLE (Najm et al., 2013). The pathophysiology of TLE has been shown to extend outside the hippocampus and it is suggested that TLE is a systems disorder with idiosyncratic cortical and subcortical abnormalities (Keller et al., 2015). Animal studies and more recently, imaging studies have implicated the piriform cortex in this regard (vide infra) (Vaughan et al., 2014).

**Fig. 1:**
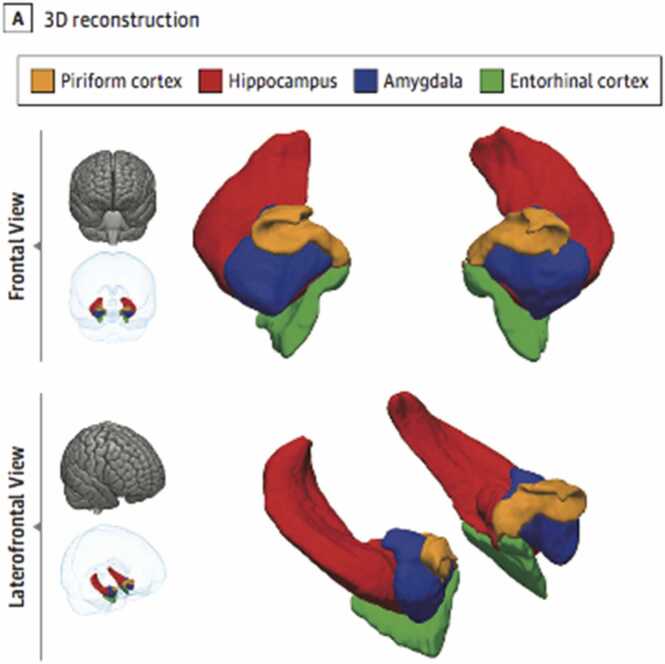
3-Dimensional reconstruction of the Piriform Cortex.

The piriform cortex is located at the confluence of the temporal and frontal lobes (Fig. 1) around the entorhinal sulcus (Gonçalves et al., 2005, Galovic et al., 2019), with frontal and temporal divisions (Young et al., 2018). The piriform cortex is three layered allocortex, as is the hippocampus (Vaughan et al., 2014). Injection of chemoconvulsants to the ‘*Area Tempestas’*of the piriform cortex, generated bilateral clonic seizures at a lower threshold than other areas of the brain (Piredda and Gale, 1985; Gale, 1988). The piriform cortex generates epileptic seizures following electrical stimulation or kindling (Löscher and Ebert, 1996, Young et al., 2018) more readily than neighbouring entorhinal cortex, amygdala and hippocampus (McIntyre and Gilby, 2008).

(adapted from Galovic et al., 2019).

The piriform cortex is affected by prolonged and repetitive seizures. Sustained seizure activity was associated with neuron loss and an increased chance of new seizure onset (Roch et al., 2002, Gonçalves et al., 2005). There were similar findings in post-mortem studies of patients who had status epilepticus (Fujikawa et al., 2000).

Activation of the piriform cortex ipsilateral to the seizure focus was seen with EEG-fMRI of inter-ictal epileptic activity. 11 C-labeled flumazenil positron emission tomography revealed reduced GABA-A receptor binding in the piriform cortex in association with greater seizure activity (Laufs et al., 2011). These and other studies (Fahoum et al., 2012, Flanagan et al., 2014) led the piriform cortex to be postulated to be a common node in networks that disseminate epileptic discharges.

The piriform cortex’s position also suggested a role as a propagation pathway of epileptic discharges in focal epilepsy, with connections to entorhinal, limbic, orbitofrontal and insular cortex, thalamus, olfactory bulb, amygdala and hippocampus (Löscher and Ebert, 1996; Vaughan et al., 2014).

There have been two volumetric analyses of the human piriform cortex using in vivo MRI Gonçalves et al. (2005) and Galovic et al. (2019). Gonçalves et al. (2005) measured piriform cortex volumes in pharmacoresistant TLE. Galovic et al. (2019) compared the extent of piriform cortex resection with surgical outcome after anterior temporal lobe resection and included the frontal component of the piriform cortex.

Gonçalves et al. (2005) reported a reduction in piriform cortex volume ipsilateral to the seizure focus, that was congruent with the degree of hippocampal, amygdaloid and entorhinal cortex volume reductions. This is in accord with the interconnectivity of these structures in the mesial temporal lobe (Vaughan et al., 2014). This questions the question of whether piriform cortex volumes would differ in TLE patients with hippocampal sclerosis (HS) and without HS.

Galovic et al. found that the chance of seizure remission after anterior temporal lobe resection was increased 16-fold if at least 50% of the piriform cortex was resected. In contrast, the volumes of resection of the hippocampus, amygdala and entorhinal cortex were not strongly correlated with post-operative seizure freedom (Galovic et al., 2019).

This led to the suggestion that the piriform cortex has a key role in seizure expression in TLE and should be identified and resected during anterior temporal lobe resection for TLE.

The objectives of this study are to implement a robust protocol for piriform cortex volumetry and determine whether there is piriform cortex atrophy in TLE, and to determine whether clinical characteristics of TLE correlate with piriform cortex volumes.

## 2 Methods

### 2.1 Subject selection and characteristics

Sixty consecutive adults with medically refractory unilateral TLE who underwent pre-surgical evaluation between 16 January 2014 and 31 October 2018 at the National Hospital for Neurology and Neurosurgery (NHNN) for consideration of anterior temporal lobe resection were included.

The diagnosis of intractable TLE was established by a multi-disciplinary epilepsy team, following MRI, video-EEG telemetry and neuropsychology profile, and FDG PET if MRI was unremarkable. Clinical data were obtained from NHNN electronic medical records (Supplementary Table 1).

27 patients had right TLE and 33 patients left TLE. At the time of MRI, the mean age of patient group was 38 yr (range: 19 – 68; SD 12.0). 31 of the 60 patients were female. Neurological insults prior to the onset of habitual epilepsy (febrile convulsions, intracranial infections, head injury) were reported in 31 (52%) of cases. 31 had hippocampal sclerosis, 18 had other focal temporal lobe pathologies (DNT, cavernoma, focal cortical dysplasia, ganglioglioma, neuroglial tumour, encephalocoele), not involving the amygdala or piriform cortex, and 11 had unremarkable MRI.

The control population comprised 20 healthy individuals (12 female) without any prior history of neurological or psychiatric illness, who had been scanned over the previous 5 years. Their mean age was 40.8 years (range: 22 – 66; SD 12.6).

The approval of the local research ethics committee was obtained for anonymised analysis of previously acquired clinical data. Healthy controls provided written consent as part of previous studies approved by the local Research Ethics Committee (IRAS 269802).

### 2.2 Image acquisition

MRI scans were acquired on a 3T GE Discovery MR750 scanner with a 32-channel coil. Sequences included a three-dimensional (3D) T1-weighted inversion-recovery fast spoiled gradient recalled echo (TE/TR/TI 3.1/7.4/400 ms, field of view (FOV) 224**×** 256**×** 256 mm, matrix 224**×** 256**×** 256, parallel imaging acceleration factor 2) and a coronal dual-echo fast recovery fast spin echo proton-density/T2-weighted (TE 30/119 ms, TR 7600 ms, FOV 220**×**220 mm, matrix 512**×**512, slice thickness 4 mm, SENSE factor 2).

### 2.3 Image alignment

Each image was rigidly realigned with a symmetric group template generated from healthy control datasets that was oriented with the hippocampal long axis along the anterior-posterior axis (Vos et. al, 2020). This template was generated using NiftyReg's iterative groupwise registration tool (Modat et. al, 2010). To remove any residual yaw in the group template, this was flipped in the left-right dimension and realigned to the mid-space of the original and left-right-flipped image.

### 2.4 MR volumetric analysis

Automated hippocampal volumetry was performed as described previously (Winston et al., 2013, Winston et al., 2017). All volumes were adjusted for total intracranial volume (TIV) (See below).

#### 2.4.1 Segmentation of the piriform cortex

Manual segmentation of the piriform cortex was carried out using ITK-Snap Version 3.8.0-beta software (Yushkevich et al., 2006). The user outlines the targeted anatomical region, and the volume of the delineated region is then automatically calculated in cubic millimetres. The MRI scans were oriented, so that coronal images were presented that were orthogonal to the long axis of the left hippocampus, and any residual yaw in the images were corrected.

**Fig. 2:**
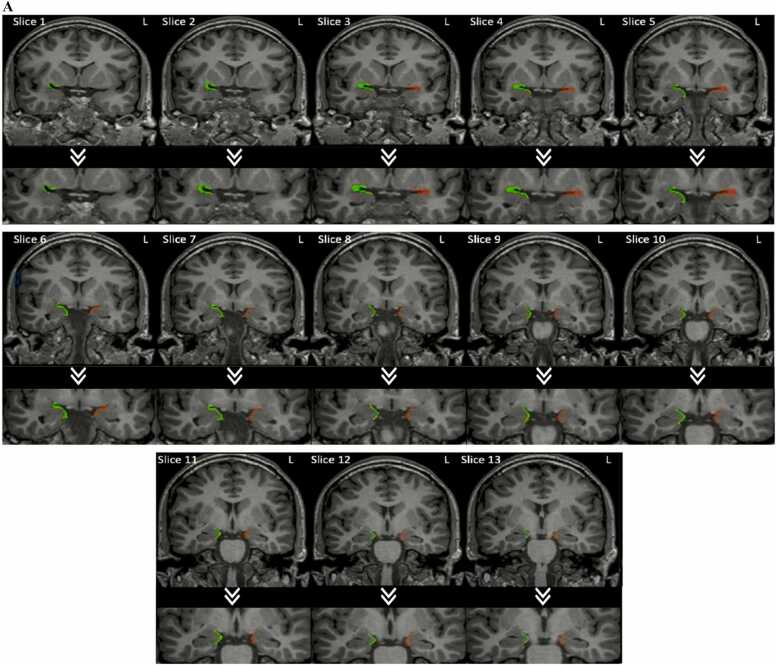

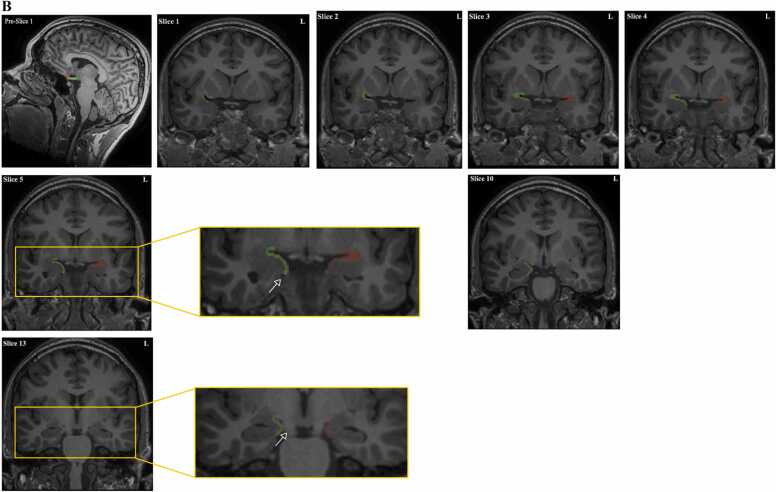
**A:** Right (Green) and Left (Red) Segmentations of Piriform Cortex in Healthy Individual on MRI. **B:** Protocol for delineating the piriform cortex. **Step 1**. *Slice 1:* The most anterior slice that includes the piriform cortex is that in which the limen insulae, the white matter tracts connecting insula and temporal lobe is evident). This marks the start of the piriform cortex and the cortical amygdala **(**PCA). On this slice, the PCA is located at the confluence of the frontal lobe and the temporal stem where it contours the inferior and superior banks of the entorhinal sulcus forming a C-shape. The frontal part projects from the core of the entorhinal sulcus and extends till 50–75% of the distance to the olfactory tubercle (Vaughan et al., 2014; Galovic et al., 2019). In pre-slice 1, the red circle is the olfactory tubercle and in the green rectangle is the optic chiasm for reference. Anatomically, the olfactory tubercle is anterior to the chiasm which can be used as a reference to locate it on sagittal MRI imaging. Following that, it was easily identified on the coronal plane and marked on the first slice. The temporal part of the piriform cortex lines the lower half of the C-shape i.e., inferior bank of the entorhinal sulcus and covers 30% of the distance to the apex of gyrus semilunaris. The apex of the gyrus semilunaris is the most prominent medial point of the gyrus. The voxel size is 1 mm isotropic. The thickness of the PCA was kept to two voxels thick in the absence of grey-white border, otherwise, it followed the grey-white interface if this was evident. The curvature of the junction between the frontal and temporal parts of the PCA always showed a clear gray-white matter differentiation laterally, therefore the thickness of this area was dependent on this differentiation across all slices. In contrast, in the portions where the PCA joins the amygdala, gray-white differentiation disappears, and the 2-voxel thickness rule applies. **Step 2**. *Slice 2:* From here, the frontal section of the PCA was maintained as above, whilst the temporal part was extended to 50% of the distance to the apex of the gyrus semilunaris. *Slice 3:* The temporal part was extended to 75% of the distance to the apex of the gyrus semilunaris on the following slice. **Step 3**. *Slice 4:* In the following slice, the temporal part of the PCA was extended to cover the gyrus semilunaris entirely, therefore representing 100% of the distance from the core of the entorhinal sulcus to the apex. This usually corresponded with the appearance of the temporal horn. However, as brain asymmetry is common, this was not the case in every patient. **Step 4**. *Slice 5:* The temporal part of the PCA was then contoured around the entire length of the gyrus semilunaris and terminated at the sulcus semiannularis. The sulcus semiannularis (*see arrow)* is denoted by a subtle dip at the lower boundary of the gyrus semilunaris. The temporal extension of the PCA to the sulcus semiannularis continues until the end slice of the piriform cortex. If the sulcus semiannularis was not clearly visible, the white matter beneath the amygdala was used to indicate the tail end of the PCA. **Step 5**. *Slice 10:* As the entorhinal sulcus shortens and the optic tracts start to move more laterally and closer to the PCA, the frontal extension becomes shorter until it disappears. This is because, as explained before, the frontal part projects from the core of the entorhinal sulcus and extends till 50–75% of the distance to the olfactory tubercle, which is kept as a constant landmark. **Step 6**. *Last Slice*: The last slice is marked as the one slice before the complete fusion of the cerebral peduncle with the pons. The thickness of the PCA was kept to 1 voxel thick here.

The piriform cortex was manually segmented using a trackball driver cursor on MRIs of all 80 study participants by two investigators (SI and JL), after training and familiarization with the protocol. The investigators were blinded as to the diagnosis and laterality of epilepsy. Each investigator outlined all 160 piriform cortices twice, in a blinded fashion. The segmentation of the left and right side was performed separately, without knowledge of the contralateral segmentation. The protocol for outlining the piriform cortex was based on anatomical points defined by histological analysis (Gonçalves et al., 2005) and utilised in another study (Galovic et al., 2019). The current study amalgamated the two algorithms with a slight modification to resolve any unclear landmarks. Both algorithms segmented the piriform cortex and the cortical amygdala (PCA) together. A MR image series exhibiting the complete segmentations of both the left and right PCA for a control subject is shown in Fig. 2A. The protocol for segmenting the left and right piriform cortex is indicated in Fig. 2B.

(adapted from Galovic et al., 2019).

#### 2.4.2 Inter-rater and intra-rater variability

Intra-rater and inter-rater reliability were assessed on all 60 TLE cases with Bland-Altman plots (Bland and Altman, 1986). For the inter-rater Bland-Altman plot, the mean difference between the volumes measured by the two investigators and 2 standard deviations of the mean difference marked the upper and lower limits of agreement. Similarly, the intra-rater test/re-test data were plotted using the initial and repeat volumetric measurements.

### 2.5 Subject and control data analysis

Data from TLE patients were subdivided into those with left TLE and right TLE for comparison with the controls. Subsequently, the subject data were split into ipsilateral and contralateral to the epileptic focus. The mean measure of the two investigators was used.

Control data were analysed first. Fitted trend lines based on least-squares linear regression model were calculated and plotted over the data points. Pearson correlation coefficients were calculated. In view of the small sample size Mann-Whitney U test was used to compare male and female control data. P-value < 0.05 was regarded as statistically significant. Findings from the control data were taken into account for the analysis of the epilepsy data. The volumes of subject piriform cortices were adjusted for intracranial volume, age and the asymmetry observed between the left and right piriform cortices.

For intracranial volume and age, the patient data were adjusted using the linear regression model which was calculated from the control data using the following equation (Free et al., 1995):

‘NV = OV - Grad (CMi - CM mean)’

NV is the corrected volume, OV is the original volume, Grad is the slope of the regression line from the control data, CMi is the measurement for the subject, and CM mean is the mean from all the control data.

We used the Mann Whitney U test to compare mean piriform cortex volumes in controls and patients with left and right TLE. Univariate and multivariate regression models were utilised to examine the relationship between MRI volumetric quantification of the piriform cortex and hippocampus with the clinical variables collected for patients with drug resistant TLE. The corrected volumes of the two anatomical structures were taken to be the ‘response variable’ and the clinical characteristics as the ‘explanatory variables’. The piriform cortex and the hippocampus were further sub-categorised into ipsilateral and contralateral volumes.

Prior to the sub-division into ipsilateral and contralateral subsets for univariate and multivariate analysis, patient data were adjusted for the asymmetry found between the left and right piriform cortices using the following equation:

‘NV=OV + (Overall Mean -Specific Side Mean)’

NV is the corrected volume, OV is original volume, overall mean is mean from all control data and specific side mean is mean from the control data of the stated side.

#### 2.5.1 Univariate analysis

The correlation between duration of epilepsy with piriform cortex and hippocampal volumes was explored, using univariate linear regression on both the dataset subcategories i.e. right/left TLE and ipsilateral/contralateral. Second, the volumes of the two structures in patients with and without previous neurological insults, and with and without secondarily generalised seizures were determined. Lastly, the volumes of the piriform cortex in those with and without hippocampal sclerosis on MRI was investigated.

Duration of epilepsy was considered the main independent variable that was used for comparing and visualizing the data for both univariate and multivariate variables and this provides intuitive visual comparisons across different variables and groups.

#### 2.5.2 Multivariate analysis

A multiple linear regression model was utilised to evaluate and distinguish the impact of each explanatory variable on piriform cortex or hippocampus volume. The model included duration of epilepsy, age of onset, seizure frequency, total number of anti-seizure medications taken as ‘explanatory variables’. For multivariate analysis with five tests on the same dataset, Bonferroni correction was applied and p < 0.01 considered significant. Statistical analyses were performed using Matlab (version R2018b).

## 3 Results

### 3.1 Inter-rater and intra-rater variability

Inter-rater (Supplementary Fig. 1), the measurements from the two investigators for the left piriform cortex shows a mean difference of 13.7 mm^3^ and a 1.96 SD of 82.95 mm^3^ and the right piriform cortex showed a mean difference of 6.85 mm^3^ and a 1.96 SD of 80.3 mm^3^. For left and right combined, the limits of agreement (1.96 SD) of 81.62 mm^3^ is 15.9% of the mean measure. The Bland Altman analysis showed no systematic error and there was good agreement between the two investigators for both the left and right piriform cortex.

Intra-rater (Supplementary Fig. 2), the initial (1) and repeat measurement (2) of investigator SI for left piriform cortex demonstrates a mean difference of 6.08 mm^3^ and a 1.96 SD of 45.56 mm^3^. The intra-rater measurements for the right piriform cortex demonstrate a mean difference of 0.3 mm^3^ and 1.96 SD of 57.64 mm^3^. For left and right combined, the limits of agreement (1.96 SD) of 53.6 mm^3^ is 10.6% of the mean. The intra-rater measurements are in good agreement with a better concordance than inter-rater measurements.

### 3.2 Control group findings

**Fig. 3:**
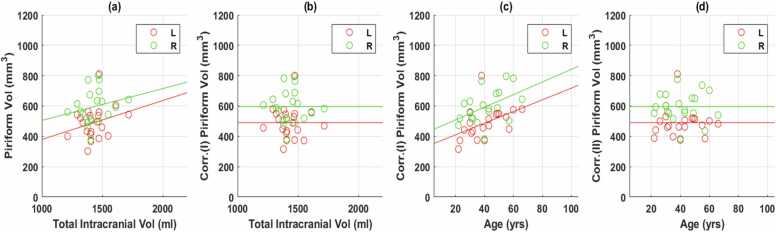
Control Data. Fig. 3a: Correlation of control data (mm^3^) with total intracranial volume. Fig. 3b: Corr. (I) mm^3^ =Corrected Metric (I) mm^3^ adjusted for intracranial volume. Fig. 3c: Correlation of control data (mm^3^) with age. Fig. 3d: Corr. (II) mm^3^ =Corrected Metric (II) mm^3^ adjusted for age and intracranial volume. L=Left, R=Right, Vol=Volume.

There was a weak positive linear correlation between intracranial volume and the left (r = 0.263) and right (r = 0.212), piriform cortex volumes (Fig. 3a) so the volumes were corrected for intracranial volume (corrected metrics I-Fig. 3b, Table 1) (Free et al., 1995). There was a positive correlation between the age of an individual and piriform cortex volumes on both left (r = 0.458, p = 0.04) and right (r = 0.483, p = 0.03) (Fig. 3c). Consequently, piriform cortex volumes were adjusted for age (corrected metrics II-Fig. 3d, Table 1).

**Table 1:** Identification of trends in control dataset:.

	**Anatomical Structures**			
	**Left Piriform Cortex**	**Right Piriform Cortex**	**Left Hippocampus**	**Right Hippocampus**
***Total Intracranial Volume*** *Correlation Coefficient, r* *p-Value* ***Corrected Metric*** *Mean* *Standard Deviation* *Range* ***Age*** *Correlation Coefficient, r* *p-Value* ***Corrected Metric*** *Mean* *Standard Deviation* *Range*				
	0.263	0.212	0.742	0.760
	0.26	0.37	**0.00**	**0.00**
	**Corrected Metric (I)**			
	490	595	2942	3008
	106	109	237	192
	315– 800	381–796	2585–3409	2642–3382

	0.458	0.483	-0.403	-0.196
	**0.04**	**0.03**	0.08	0.41
	**Corrected Metric (II)**		**Corrected Metric (I)**	
	490	595	2942	3008
	94.0	95.1	237	192
	377–811	385–776	2585–3409	2642–3382

The mean right piriform cortex volume was 17.7% larger than the left piriform cortex (Table 2). The mean control volume for the right piriform cortex (corrected metrics II) was 595 + /- 95.1 mm^3^ [mean + /-SD]; left, 490 + /− 94.0 mm^3^ [mean + /-SD] (Table 2). In controls, the right piriform cortex extended by a mean of 6 mm (Range: −4 to 12; SD: 4.6) more anteriorly than the left piriform cortex (Supplementary table 2). This asymmetry was taken into account when the patient dataset was re-categorised into ipsilateral and contralateral subsets for analysis (Supplementary Tables 3, 4).

**Table 2:** Piriform cortex volumes corrected for total intracranial volume & age (corrected metric II) and hippocampus volumes corrected for total intracranial measurements (corrected metric I).

	**Structure Volumes (mm**^**3**^**)**			
***Derivation Cohort***	**Left Piriform Cortex:**	**Right Piriform Cortex:**	**Left Hippocampus:**	**Right Hippocampus:**
***Controls*** Mean, * % ∆ *R-L (p value)* *Standard Deviation* *Range*	**Corrected Metric (II)**		**Corrected Metric (I)**	
	
	490	595 * 17.7 (p = 0.00)	2942	3008
	94.0	95.1	237	192
	377–811	385–776	2585–3409	2642–3382
***Left Sided TLE*** Mean, * % ∆ *R-L (p value)* *%* ∆ *with Controls (p value)* *SD* *Range*				
	465	601 * 22.6 (p = 0.00)	2395	2917
	-5.1 (0.48)	-1 (0.92)		
	99	110	539	287
	223–704	356–849	1226–3317	2166–3718
***Right Sided TLE*** Mean, * % ∆ *R-L (p value)* *%* ∆ *with Controls (p value)* *SD* *Range*				
	477	582 * 18%(p = 0.00)	2951	2495
	-2.7 (0.94)	-2.2 (0.71)		
	115	86	276	537
	188–664	417–709	2263–3433	1589–3514
* % ∆ *R-L (p value =percentage difference between right and left piriform cortex (p value)* *%* ∆ *with Controls (p value) = percentage difference with controls (p value)*				

### 3.3 Piriform cortex volumes in temporal lobe epilepsy

#### 3.3.1 Individuals with left TLE

The right piriform cortex was a mean 22.6% (p < 0.001) larger than the left (Table 2). The corrected volume (II) of the ipsilateral piriform cortex was 465 + /− 99 mm^3^ [mean+ /-SD] (Table 2). This was not significantly different from the left mean piriform cortex volume in controls (Table 2). The corrected contralateral, right, mean piriform cortex volume was 601 + /− 110 mm^3^ [mean+ /-SD]. This was not significantly different from the mean right control piriform cortex volume (Table 2).

#### 3.3.2 Individuals with right TLE

The right piriform cortex in right TLE was a mean 18% (p < 0.001) larger than the left (Table 2). The corrected volume (II) of the ipsilateral piriform cortex was 582 + /- 86 mm^3^ [mean+ /-SD] (Table 2). This was not significantly different from the right piriform cortex mean volume in controls (Table 2). The corrected contralateral, left, piriform cortex mean volume was 477 + /- 115 mm^3^ [mean+ /-SD] (Table 2). This was not significantly different from the mean left control piriform cortex volume (Table 2).

### 3.4 Correlation of piriform cortex & hippocampus volumes with clinical features

**Fig. 4:**
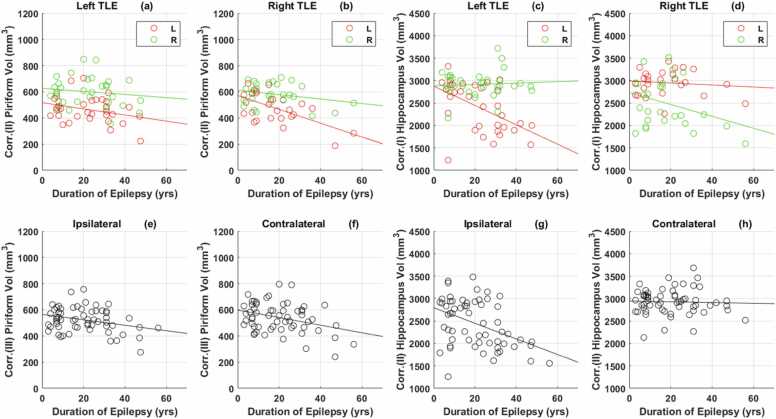
**Correlation of subject data with duration of epilepsy.** Fig. 4***a, b**: Corr. (II) Piriform Vol = Corrected (II) piriform cortex volume adjusted for age and intracranial volume*. Fig. 4***c, d**: Corr. (I) Hippocampus Vol = Corrected (I) hippocampus volume adjusted for intracranial volume*. Fig. 4***e, f:** Corr.(III) Piriform Vol =Corrected (III) piriform cortex volume adjusted for age, intracranial volume and asymmetry*. Fig. 4***g, h:** Hippocampus Vol= Corrected (II) hippocampus volume adjusted for intracranial volume and asymmetry*. *L=Left, R =Right*.

Supplementary Tablesmetry of piriform cortex volumes (Fig. 4a,b), analyses were carried out on piriform cortex volumes corrected for age, intracranial volumes and asymmetry (corrected metrics III-Supplementary Table 3).

#### 3.4.1 Duration of epilepsy

The ipsilateral (r = −0.291, p = 0.01) and contralateral (r = −0.338, p = 0.01) piriform cortices’ volumes were smaller with a longer duration of epilepsy (Fig. 4e,f). The ipsilateral hippocampi were smaller with a longer duration of epilepsy (r = −0.424, p < 0.001) (Fig. 4 g). The volume of the contralateral hippocampus was not significantly correlated with the duration of epilepsy (r = −0.039, p = 0.77) (Fig. 4 h).

#### 3.4.2 Prior neurological insults

The ipsilateral piriform cortex did not show an effect and the contralateral piriform cortex showed a decrease in volume of marginal significance (r = −0.356, p = 0.05) (Supplementary Fig. 3a-d, Supplementary Table 3). The ipsilateral hippocampus (r = −0.479, p = 0.01) was smaller if there had been a prior neurological insult (Supplementary Fig. 3a-d, Supplementary Table 3). The contralateral hippocampus did not show this effect (r = −0.066, p = 0.72) (Supplementary Fig. 3a-d, Supplementary Table 3).

#### 3.4.3 Generalized seizures

The occurrence of generalized seizures demonstrated no impact on ipsilateral (r = −0.287, p = 0.06) or contralateral (r = −0.050, p = 0.74) hippocampal volumes nor ipsilateral piriform cortex (r = −0.189, p = 0.21) (Supplementary Fig. 3e-h, Supplementary Table 3). The contralateral piriform cortex was smaller in those with generalized seizures of marginal significance (r = −0.301, p = 0.05) (Supplementary Fig. 3e-h, Supplementary Table 3).

#### 3.4.4 Hippocampal sclerosis

31 patients had hippocampal sclerosis. The ipsilateral (r = −0.418, p = 0.02) and contralateral (r = −0.443, p = 0.01) piriform cortex volumes were smaller in those with hippocampal sclerosis (Supplementary Fig. 3i-j, Supplementary Table 3). There was not a statistically significant difference in mean ipsilateral or contralateral piriform cortex volumes between TLE patient groups with and without HS.

### 3.5 Multivariate Analysis

A longer duration of epilepsy was associated with smaller ipsilateral (Beta=−0.490, p = 0.002) and contralateral piriform cortices (Beta=−0.556, 0.001) and a smaller ipsilateral hippocampus (Beta=−0.537, p = 0.001) (Supplementary Fig. 4, Supplementary Table 4).

There were no statistically significant correlations between the piriform cortex or hippocampal volumes and the age of onset, reported seizure frequency reported, the number of antiseizure medications taken at present or in the past (Supplementary Fig. 4a-p, Supplementary Table 4).

## 4 Discussion

The principal findings of this investigation were:

• The right piriform cortex was larger and extended more anteriorly than the left piriform cortex in controls and those with left and right TLE.
• In TLE patients overall, the piriform cortices were not significantly smaller than in controls.
• Hippocampal sclerosis was associated with decreased ipsilateral and contralateral piriform cortex volumes.
• Ipsilateral and contralateral piriform cortex volumes were smaller with a longer duration of epilepsy.
• Prior neurological insults and generalized seizures were associated with marginally smaller contralateral piriform cortex volumes.
• There was no significant association between piriform cortex volumes and the frequency of focal seizures with impaired awareness or the number of anti-seizure medications taken at present or ever.

### 4.1 Left-right asymmetry of the piriform cortex

The right piriform cortex extended more anteriorly than the left and was significantly larger in controls and TLE. This was not due to technical factors such as image yaw or the segmentation method as we corrected the former and verified that flipping images did not affect results. Importantly, the left and right piriform cortices were outlined separately, without knowledge of the first outlining, so the outlining of the second piriform cortex would not be influenced by the first. Previous studies on volumetric measurement of the piriform cortex did not report significant left-right asymmetry between the two sides (Gonçalves et al., 2005; Galovic et al., 2019) and did not mention whether the left and right segmentations of the piriform cortices were done separately or not.

This finding is in keeping with the asymmetry of Meyer’s loop of the optic radiation (Yogarajah et al., 2009, Nowell et al., 2015). The mean distance from the temporal pole to the anterior margin of Meyer’s loop was 45.5 mm on the right and 39.7 mm on the left (Dreessen et al., 2014). We infer that the piriform cortex asymmetry is a consequence of Yakovlevian torque, the tendency of the right side of the human brain to be warped slightly forward relative to the left (Lecours, 1989).

### 4.2 Piriform cortex volumes in TLE

The finding of no significant reduction of mean piriform cortex in TLE overall, compared to controls, contrasts with Galovic’s finding of bilateral reduction for right-sided TLE and ipsilateral reduction for left-sided TLE. Goncalves et al. in 2005, reported a reduction in ipsilateral but not contralateral piriform cortex volume in TLE. The current study, however found smaller piriform cortices in those with hippocampal sclerosis. In the Galovic study 86/107 had HS, and the higher proportion of HS in that study is the likely explanation for the variation in finding. Given this, it would be appropriate in future studies to further explore piriform cortex volumetry in TLE patients with and without HS.

### 4.3 Piriform volumetry protocols

The Goncalves protocol did not include the frontal lobe portion of the piriform cortex. Both Goncalves and Galovic used the peri-rhinal cortex thickness as a guide to define the thickness of the piriform cortex for the first three slices, which differed from the protocol used in this study. Moreover, the determination of the most posterior slice of piriform cortex segmentation varied between all three studies. Goncalves marked the 'opening of the hippocampus fissure’ as the end slice. Galovic marked the ‘appearance of the interpeduncular cistern and mammillary bodies’ as the end slice. In this study, the complete fusion of the cerebral peduncle indicated the most posterior slice of the piriform cortex. The variance between the three protocols and the MR image acquisition may explain the differences in the volumetric measures of the piriform cortex between the studies.

### 4.4 Age and piriform cortex volumes in controls

Ageing is associated with cerebral atrophy (Peters, 2006, Esiri, 2007). The finding of larger piriform cortex volume with age in control data was not anticipated. Sulcal morphology changes with age, being wider in older individuals (Jin et al., 2018). It is possible that the widening and increase in the indentation of sulci associated with age-related atrophy might affect the manual tracing of the piriform cortex around the entorhinal sulcus (Vaughan et al., 2014) and a wider entorhinal sulcus effectively increases the surface area of the piriform cortex outlined.

### 4.5 Piriform cortex volumes and hippocampal sclerosis

Hippocampal sclerosis and ipsilateral hippocampal atrophy were associated with bilateral piriform atrophy, which has not been described previously. It is recognized that the pathophysiology in mesial TLE extends beyond the hippocampus and the amygdala (Salmenpera et al., 2000, Jutila et al., 2001, Bernasconi et al., 2003; Alhusaini et al., 2012). In TLE, diffusion weighted MRI studies have illustrated a variation in bilateral temporal as well as extra-temporal pathways with a reduction in axonal density (Winston et al., 2020). Specifically, TLE with HS has been evidenced to have more widespread damage than TLE without HS in several studies (Sone et al., 2018). In a study evaluating the histological ultrastructure of a subset of patients with HS TLE against non-HS TLE, there was reduction in the temporopolar axonal density and axonal diameter in those with HS TLE. (Demerath et al., 2021).

### 4.6 Piriform cortex volumes and clinical characteristics

We observed a correlation between a longer duration of epilepsy and smaller ipsilateral and contralateral piriform cortex volumes. The duration of epilepsy is a proxy for the impact of the condition, and the total lifetime seizure burden will vary between patients. Together with our observation that bilateral piriform atrophy is associated with unilateral hippocampal sclerosis, our findings are in keeping with a recent meta-analysis that a longer duration of epilepsy and increased seizure frequency were associated with a decrease in ipsilateral hippocampal volume (Caciagli et al., 2017). We infer that progressive bilateral damage of the piriform cortex occurs as a consequence of refractory unilateral TLE.

The current study found the contralateral piriform cortex volume to be marginally smaller in those with secondarily generalised seizures, and a history of a prior neurological insult. This finding was of only borderline significance and raises the question of the functional connectivity between the temporal lobe and contralateral piriform cortex.

The piriform cortex volume was not associated with seizure frequency. Similarly, Goncalves et al., 2005 did not find any correlation between piriform cortex volumes and lifetime reported seizure frequency. The current study did not find any correlation between piriform cortex volumes and the number of anti-seizures medications taken. This concurs with previous longitudinal studies looking at the effect of anti-seizure drugs and regional atrophy (Liu et al., 2005, Salmenpera et al., 2005).

### 4.7 Methodological considerations

#### 4.7.1 Strengths

The rigorous methodology for the manual segmentation of the piriform cortex is reflected by good inter-rater (limit of agreement 15.9%) and intra-rater (limit of agreement 10.6%) reliability. Multiple steps were taken to reduce any investigator bias, including thorough training of investigators. The segmentations were performed in a random order with the left and right sides outlined independently of each other by two separate blinded investigators.

Measures were taken to ensure accuracy of the volumetric measurements of piriform cortex, including the re-slicing of MR images to correct for any yaw as well as the left-to-right flipping of MR images, so that both the left and right segmentations were done on the right side. The flipping of MR images excluded asymmetric bias which may result from laterality of visual perception (Maltbie et al., 2012).

#### 4.7.2 Limitations

A limitation of MR volumetric studies of the anterior medial temporal lobe is the lack of overt anatomical landmarks. The piriform cortex thickness is difficult to delineate due to the lack of any landmarks. The thickness was defined as up to the white matter where there was a grey matter/white matter differentiation and was kept to 2 voxels thick where there was no evident boundary between the piriform cortex and amygdala. The MR images were oriented with the hippocampal long axis along the anterior-posterior axis similar to the previous two studies on MR volumetry of the piriform cortex. However, the ‘piriform axis’ is an oblique-axial orientation and may be better for measuring the piriform cortex volume as it diminishes partial voluming (Vaughan et al., 2014, Young et al., 2019). A cross-sectional design is pragmatic but lacks the sensitivity of a longitudinal design to evaluate the impact of clinical factors on piriform cortex volumes.

## 5 Conclusion

The finding of the asymmetry of the piriform cortex adds to understanding of the asymmetry of brain development. The finding of bilateral piriform cortex atrophy, the extent of which relates to the duration of the epilepsy is concordant with the concept that the piriform cortices are involved in the pathophysiology of temporal lobe epilepsy.

A prospective longitudinal neuro-imaging study comprising a larger patient population as well as control population to analyse the progression of the piriform cortex volume during the course of epilepsy and the correlation with clinical variables is warranted.

Given the greater chances of seizure freedom after anterior temporal lobe resection for drug refractory TLE, if > 50% of the piriform cortex was removed (Galovic et al., 2019), the segmentation of the piriform cortex on preoperative MRI, and the inclusion of the temporal portion in anterior temporal lobe resection for drug-refractory mesial TLE should be considered. To facilitate this, the development of automated segmentation, that is specific for left and right piriform cortices and which is necessary in view of the asymmetry, is in progress.

## Declaration of Competing Interest

None.
