# Supplementary material for: Volumetric analysis of the piriform cortex in temporal lobe epilepsy
Source: Epilepsy Res. 2022 Sep;185:106971. doi: 10.1016/j.eplepsyres.2022.106971 (PMC10510027; doi:10.1016/j.eplepsyres.2022.106971)
Supplement: Supplementary file 1 — Supplementary material [file mmc1.docx]

### SUPPLEMENTARY MATERIAL

**Supplementary Table 1:** Summary of the collected patient demographics and clinical variables.

| ***Patient Demographics and Clinical Variables*** | |
| --- | --- |
| **Clinical Variables** |  |
| **Age, median (IQR) yr** | 36.5 (29.5-47.5) |
| **Gender, No. (%)**  **Male**  **Female** |  |
|  | 29 (48.3) |
|  | 31 (51.7) |
| **Duration of Epilepsy at MR Scan, median (IQR), yr** | 19.5 (8-31) |
| **Age of Onset, median (IQR) yr** | 16 (8.5-25) |
| **Seizures, No. (%)**  **Only CPS**  **CPS & Secondary Generalised Seizures**  **CPS & Status Epilepticus** |  |
|  | 9 (15.0) |
|  | 45 (75.0) |
|  | 6 (10.0) |
| **CPS Frequency Per Month, median (IQR)** | 8 (3-15) |
| **MRI Findings, No. (%)**  **Hippocampal Sclerosis**  **Others:** DNT, cavernoma, focal cortical dysplasia, ganglioma, neuroglial tumour, encephalocoele  **Normal** |  |
|  | 31 (51.7) |
|  | 18 (30.0) |
|  | 11 (18.3) |
| **Prior Neurological Insults, No. (%)**  **Positive**  **Negative**  **Unknown** |  |
|  | 29 (48.3) |
|  | 28 (46.7) |
|  | 3 (5.00) |

FIA: focal seizures with impaired awareness

DNT: dysembryoplastic neuroepithelial tumor

**Supplementary Table 2:** The analysis of left/right piriform cortex asymmetry in controls

| **Controls** | **Piriform Cortex Asymmetry** | | | |
| --- | --- | --- | --- | --- |
|  | *Anterior Portion (R-L) Slices* | *Anterior Portion (R-L) mm3* | *Posterior Portion (R-L)*  *Slices* | *Posterior Portion (R-L)*  *mm3* |
| *1* | 0 | 0 | 0 | 0 |
| *2* | 2 | 8 | 0 | 0 |
| *3* | 3 | 12 | 0 | 0 |
| *4* | 2 | 8 | 0 | 0 |
| *5* | 1 | 4 | 1 | 4 |
| *6* | 0 | 0 | 0 | 0 |
| *7* | 0 | 0 | 1 | 4 |
| *8* | 2 | 8 | 1 | 4 |
| *9* | 3 | 12 | -1 | -4 |
| *10* | 2 | 8 | -1 | -4 |
| *11* | 2 | 8 | 0 | 0 |
| *12* | 2 | 8 | 1 | 4 |
| *13* | 1 | 4 | 0 | 0 |
| *14* | -1 | -4 | 1 | 4 |
| *15* | 2 | 8 | -2 | -8 |
| *16* | 3 | 12 | 0 | 0 |
| *17* | 0 | 0 | 1 | 4 |
| *18* | 2 | 8 | 0 | 0 |
| *19* | 2 | 8 | 0 | 0 |
| *20* | 2 | 8 | 0 | 0 |
| *Mean* | 1.5 | 6 | 0.1 | 0.4 |
| *Standard Deviation* | 1.15 | 4.585 | 0.79 | 3.15 |
| *Range* | -1 to 3 | -4 to 12 | -2 to 1 | -8 to 4 |

| **Supplementary Table 3:** Univariate analysis using piriform cortex volumes corrected for age, total intracranial volume and systematic right bias (corrected metric III) and hippocampus volumes corrected for age and systematic right bias (corrected metric II): The role of clinical variables influencing volume of the piriform cortex and the hippocampus in patients with TLE | | | | | | | | |
| --- | --- | --- | --- | --- | --- | --- | --- | --- |
|  | **Implicated Anatomical Structure** | | | | | | | |
|  | **Piriform Cortex: Ipsilateral** | | **Piriform Cortex: Contralateral** | | **Hippocampus: Ipsilateral** | | **Hippocampus: Contralateral** | |
| ***Corrected Metric*** | ***Corrected Metric (III)*** | | | | ***Corrected Metric (II)*** | | | |
| *Mean*  *SD*  *Range* | 523 | | 540 | | 2443 | | 2929 | |
|  | 92 | | 112 | | 534 | | 284 | |
|  | 276-757 | | 241-796 | | 1259-3481 | | 2133-3685 | |
| ***Duration of Epilepsy***  *r*  *p-Value* |  | | | | | | | |
|  | -0.291 | | -0.338 | | -0.424 | | -0.039 | |
|  | **0.01** | | **0.01** | | **0.00** | | 0.77 | |
| ***Prior Neurological Insults***  *r*    *p-Value* |  | | | | | | | |
|  | Y | -0.292 | Y | -0.356 | Y | -0.479 | Y | -0.066 |
|  | N | -0.296 | N | -0.318 | N | -0.348 | N | -0.007 |
|  | Y | 0.11 | Y | **0.05** | Y | **0.01** | Y | 0.72 |
|  | N | 0.13 | N | 0.10 | N | 0.07 | N | 0.97 |
| ***Hx Secondary Gen.Seizures***  *r*    *p-Value* |  | | | | | | | |
|  | Y | -0.189 | Y | -0.301 | Y | -0.287 | Y | -0.050 |
|  | N | -0.344 | N | -0.340 | N | -0.807 | N | 0.003 |
|  | Y | 0.21 | Y | **0.05** | Y | 0.06 | Y | 0.74 |
|  | N | 0.21 | N | 0.22 | N | 0.06 | N | 0.99 |
| ***MRI Findings***  *r*    *p-Value* |  | | | | | | | |
|  | N-HS | 0.058 | N-HS | -0.149 | N-HS | -0.141 | N-HS | 0.368 |
|  | HS | -0.418 | HS | -0.443 | HS | -0.381 | HS | -0.090 |
|  | N-HS | 0.77 | N-HS | 0.44 | N-HS | 0.47 | N-HS | **0.05** |
|  | HS | **0.02** | HS | **0.01** | HS | **0.04** | HS | 0.63 |
| *r=Correlation Coefficient.*  *Y=Yes (Presence of Prior Neurological Insult/Secondary Generalised Seizures/Status Epilepticus), N=No (Absence of Prior Neurological Insult/Secondary Generalised Seizures/Status Epilepticus), HS=Hippocampal Sclerosis, N-HS = Non-Hippocampal Sclerosis* | | | | | | | | |

| **Supplementary Table 4:** Multivariate Analysis using piriform cortex volumes corrected for age, total intracranial volume and systematic right bias (corrected metric III) and hippocampus volumes corrected for age and systematic right bias (corrected metric II): Clinical variables influencing volume of the piriform cortex and the hippocampus in patients with TLE | | | | |
| --- | --- | --- | --- | --- |
|  | **Anatomical Structure** | | | |
|  | **Piriform Cortex: Ipsilateral** | **Piriform Cortex: Contralateral** | **Hippocampus: Ipsilateral** | **Hippocampus: Contralateral** |
| Adjusted R^2^  p-Value | 0.134 | 0.152 | -0.157 | -0.042 |
|  | 0.02 | 0.01 | 0.01 | 0.76 |
| **Duration of Epilepsy**  *Beta*  *p-Value* |  | | | |
|  | -0.490 | -0.556 | -0.537 | -0.064 |
|  | **0.002** | **0.001** | **0.001** | 0.70 |
| **Age at Onset**  *Beta*  *p-Value* |  | | | |
|  | -0.382 | -0.288 | -0.135 | 0.040 |
|  | 0.01 | 0.05 | 0.34 | 0.80 |
| **Seizure Frequency**  *Beta*  *p-Value* |  | | | |
|  | 0.023 | 0.098 | 0.119 | 0.034 |
|  | 0.85 | 0.43 | 0.34 | 0.81 |
| **No. of Med. (All)**  *Beta*  *p-Value* |  | | | |
|  | 0.018 | 0.173 | 0.105 | 0.090 |
|  | 0.89 | 0.19 | 0.43 | 0.54 |
| **No. of Med. (Current)**  *Beta*  *p-Value* |  | | | |
|  | -0.092 | -0.216 | -0.166 | -0.217 |
|  | 0.48 | 0.10 | 0.20 | 0.13 |
| *P-Values < 0.01 were statistically significant (Bonferroni Correction)* | | | | |

**Supplementary Fig. 1**. Scatter plots illustrating inter-rater variability of measurements of the left and right piriform cortex of 60 patients


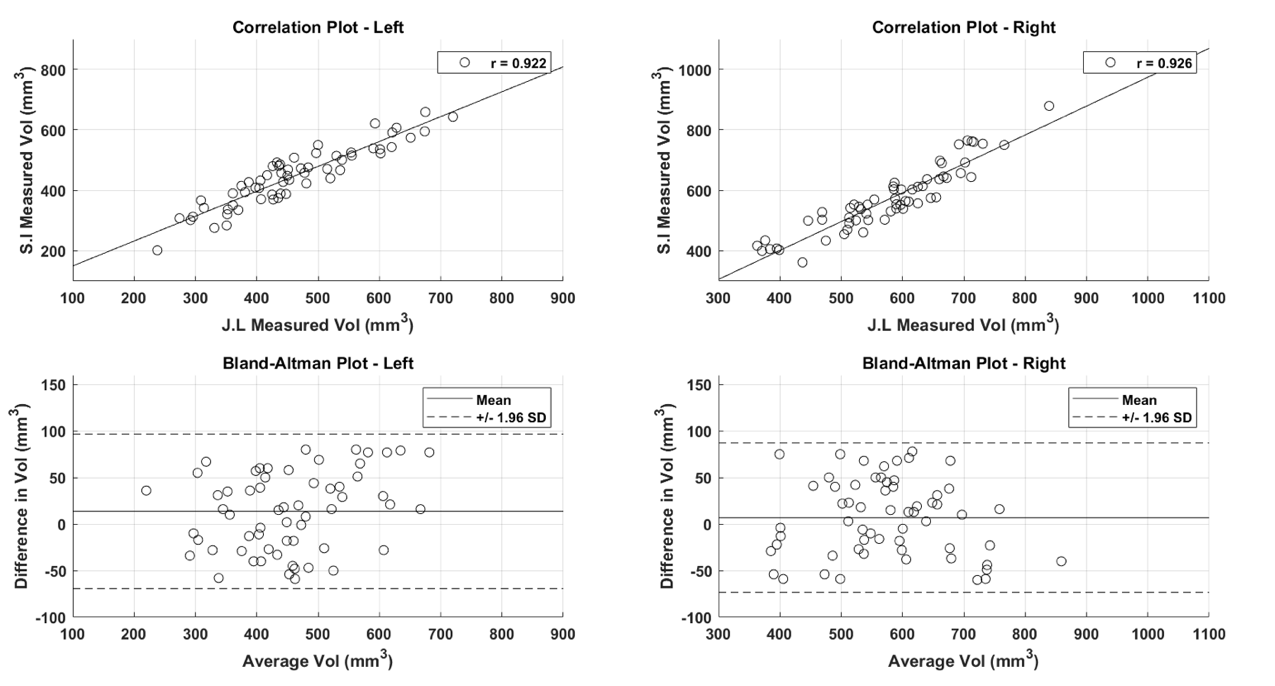


**Supplementary Figure 2:** Scatter plots illustrating intra-rater variability of initial (I) and repeat (II) measurements of the left and right piriform cortex of 60 patients

***
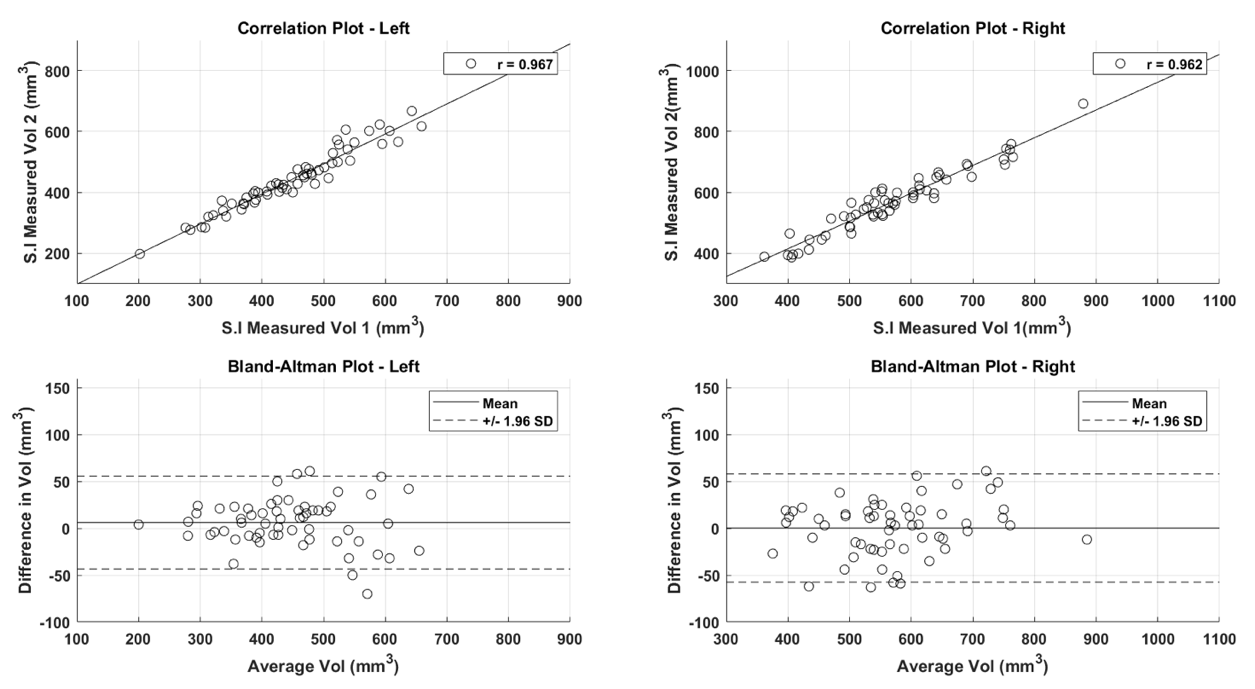
***


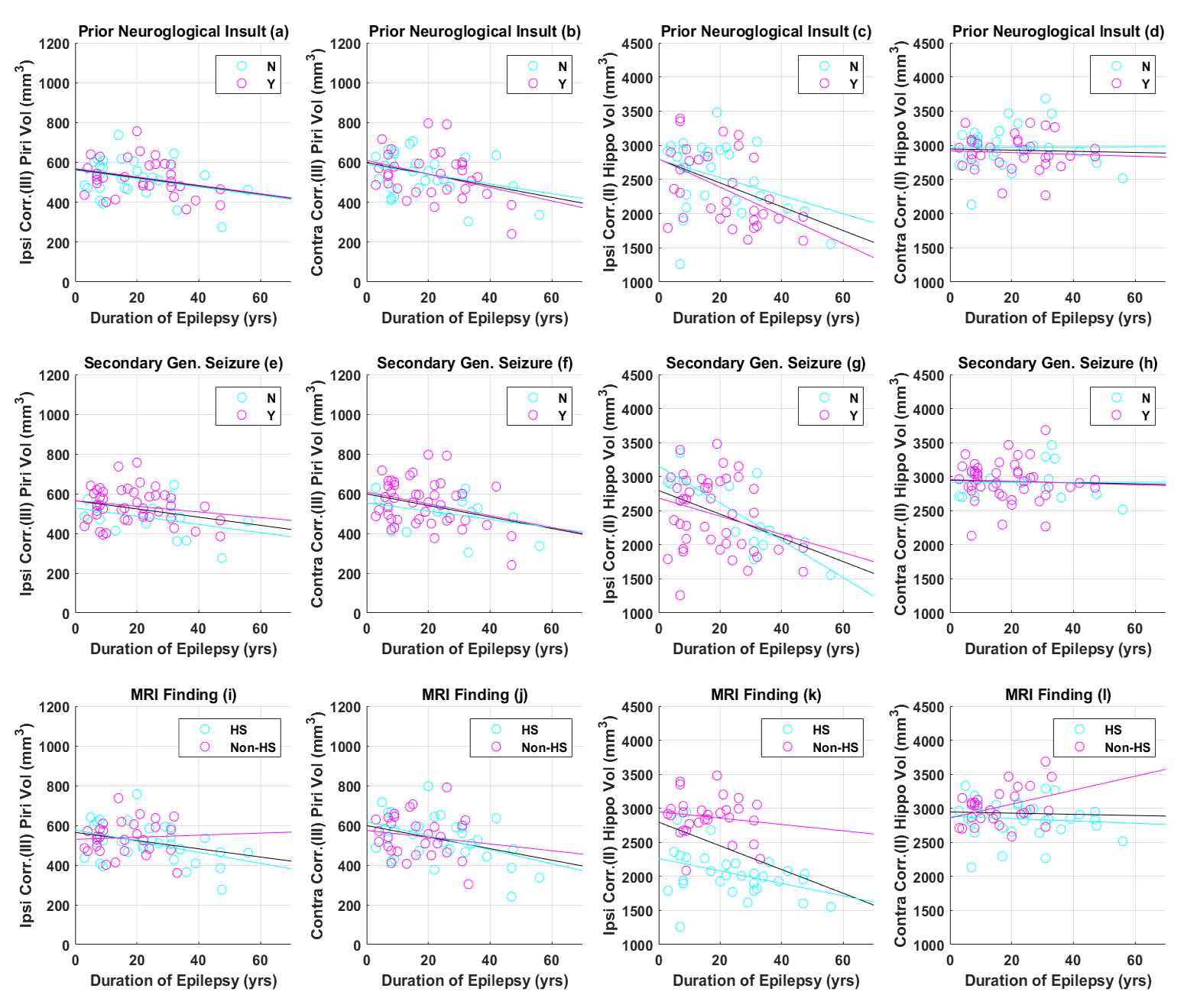
**Supplementary Fig. 3:** Univariate Analysis

*Ipsi=Ipsilateral, Contra=Contralateral, Corr. =Corrected, Piri =Piriform Cortex, Hippo=Hippocampus, Vol=Volume. Y=Yes (Presence of Prior Neurological Insult/Secondary Generalised Seizures), N=No (Absence of Prior Neurological Insult/Secondary Generalised Seizures), HS=Hippocampal Sclerosis, N-HS = Non-Hippocampal Sclerosis*


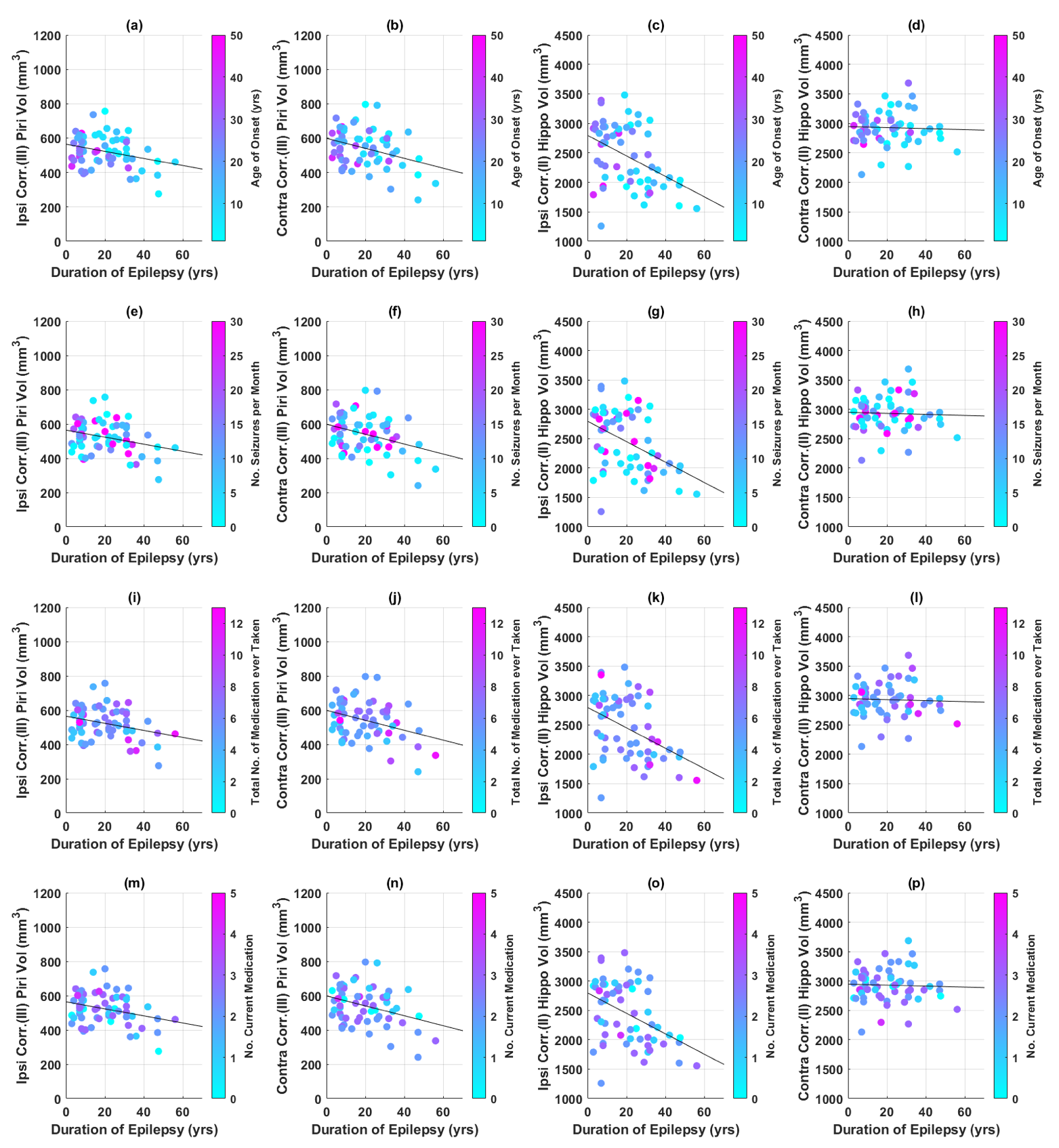
**Supplementary Fig. 4:** Multivariate Analysis

*Ipsi=Ipsilateral, Contra=Contralateral, Corr. =Corrected, Piri =Piriform Cortex, Hippo=Hippocampus, Vol=Volume*
